# Epilepsy Prediction and Detection Using Attention-CssCDBN with Dual-Task Learning

**DOI:** 10.3390/s25010051

**Published:** 2024-12-25

**Authors:** Weizheng Qiao, Xiaojun Bi, Lu Han, Yulin Zhang

**Affiliations:** 1 Laboratory of Ethnic Language Intelligent Analysis and Security Governance of MOE, Minzu University of China, Beijing 100081, China; qiaoweizheng@hrbeu.edu.cn (W.Q.);; 2 College of Information Engineering, Minzu University of China, Beijing 100081, China

**Keywords:** epilepsy prediction and detection, electroencephalograms, deep learning, convolutional deep belief network, Transformer, dual-task learning

## Abstract

Epilepsy is a group of neurological disorders characterized by epileptic seizures, and it affects tens of millions of people worldwide. Currently, the most effective diagnostic method employs the monitoring of brain activity through electroencephalogram (EEG). However, it is critical to predict epileptic seizures in patients prior to their onset, allowing for the administration of preventive medications before the seizure occurs. As a pivotal application of artificial intelligence in medical treatment, learning the features of EEGs for epilepsy prediction and detection remains a challenging problem, primarily due to the presence of intra-class and inter-class variations in EEG signals. In this study, we propose the spatio-temporal EEGNet, which integrates contractive slab and spike convolutional deep belief network (CssCDBN) with a self-attention architecture, augmented by dual-task learning to address this issue. Initially, our model was designed to extract high-order and deep representations from EEG spectrum images, enabling the simultaneous capture of spatial and temporal information. Furthermore, EEG-based verification aids in reducing intra-class variation by considering the time correlation of the EEG during the fine-tuning stage, resulting in easier inference and training. The results demonstrate the notable efficacy of our proposed method. Our method achieved a sensitivity of 98.5%, a false-positive rate (FPR) of 0.041, a prediction time of 50.92 min during the epilepsy prediction task, and an accuracy of 94.1% during the epilepsy detection task, demonstrating significant improvements over current state-of-the-art methods.

## 1. Introduction

Epilepsy, a chronic neurological disorder characterized by recurrent seizures, has impacted individuals across the globe and gained considerable research attention. Accurate diagnosis, which is vital for effective treatment and management, frequently depends on electroencephalogram (EEG). The EEG is a non-invasive technique that records the brain’s electrical activity over time, providing crucial insights into the disorder. It can detect abnormal patterns indicative of seizure activity or epileptiform discharges. These patterns assist clinicians in identifying specific features such as spike-and-wave complexes, sharp waves, and slow wave activity, all characteristic of epileptic activity.

However, current clinical practice relies heavily on experienced neurologists to identify characteristic patterns in EEGs. This approach is beset with several limitations. Firstly, the visual analysis of EEG data is an arduous task, necessitating trained professionals to dedicate hours to scrutinizing a patient’s daily recordings. This level of resource allocation is commonly unavailable in developing countries [1]. Secondly, the ability to predict epileptic seizures with accuracy can provide invaluable early warnings, enabling prompt medical intervention. This predictive capability is especially critical in managing epilepsy, a condition characterized by unpredictable and potentially dangerous seizures.

EEG signals are inherently complex, exhibiting high-dimensional, nonlinear characteristics with significant variations across individuals. These attributes pose additional challenges for automatic recognition algorithms, which strive to accurately interpret and classify EEG data [2]. Particularly, cross-patient classification remains an urgent problem. The extraction of spatial-temporal features from EEG signals is essential for accurate classification. Therefore, extracting spatial-temporal features from EEG signals continues to be a significant challenge. 

In light of these limitations and challenges, we aimed to develop a novel automated method that is effective for both seizure prediction and detection tasks. The convolutional deep belief network (CDBN) is widely recognized as a deep probabilistic graphical model, demonstrating notable proficiency in learning abstract representations and achieving remarkable results across various domains [3,4,5]. It encompasses a diverse array of training methodologies [6]. The CDBN is comprised of multiple convolutional restricted Boltzmann machines (CRBMs), which are designed to extract translation-invariant features from images [7,8,9]. This approach is inspired by the architecture of convolutional neural networks (CNNs) [10], leveraging their ability to capture spatial hierarchies in visual data. Although discussions on CRBMs have waned recently due to the significant advancements made in CNNs for diverse vision applications, this paper attempts to exploit the advantages of this model for the task of EEG signal classification. Obviously, the direct application of CDBN to epilepsy diagnosis is not feasible. CDBN is specifically composed of CRBM, and the ability of CRBM to learn EEG features significantly affects the modeling capacity of CDBN [11]. Consequently, enhancing the capabilities of CRBMs proves to be of considerable importance. Nonetheless, the traditional CDBN exhibits inadequate feature extraction and limited robustness to variations. Specifically, the CDBN disregards the temporal correlations inherent in EEG signals, leading to poor learning ability, consistent with intra-class differences. Furthermore, incorporating these insights has the potential to significantly improve the performance of epilepsy prediction and detection.

To address these issues, this paper proposes a novel probability graph model that integrates a self-attention mechanism and employs dual-task learning. Our hypotheses are as follows:

1. Enhanced Feature Extraction: By optimizing the energy function of CRBM and incorporating an element-wise multiplication operation between slab and spike units [12,13], we hypothesize that the feature extraction capabilities within the receptive field will be significantly enhanced, leading to improved performance in epilepsy detection and prediction;
2. Robustness to Variations: Through the adoption of a contractive term of probabilistic version for regularization, we hypothesize that the learning representations will be more robust to the variations of input EEG data, inducing a mapping relationship that intensely contracts the training data. Then, we can construct the contractive slab and spike convolutional deep belief network (CssCDBN) by stacking multiple trained CssCRBMs, which are further devoting to learning hierarchical and abstract representations. While related motivations for the model were published in our previous work [14], we extend them into CDBN and attempt them for the first time for epilepsy detection and prediction application;
3. Contextual Temporal Feature Focus: By integrating a self-attention mechanism, we hypothesize that the model will be able to capture intricate dependencies and relationships over time within EEG signals, improving its ability to detect and predict epileptic seizures;
4. Dual-Task Learning Benefits: Employing a dual-task learning framework [15], we hypothesize that the model will enhance inter-class variations while minimizing intra-class variations, further improving its performance in epilepsy diagnosis.

The remainder of this paper is organized as follows. Section 2 reviews the related work from the literature. Section 3 provides the details of our proposed approach with complete inferences. Section 4 analyzes the experimental results. Our further discussion is provided in Section 5. Section 6 presents our conclusions.

## 2. Literature Review and Current Method

The related works on this issue have focused on the design of the human-experience features of seizure manifestations in EEG [16,17,18] over the past years. As a representative method belonging to conventional algorithms, Cho D et al. proposed an improved SVM with phase-locking values as features [19]. Alotaiby et al. conducted seizure prediction by adopting CSP and LDA, considering the common special pattern statistics [20]. In [21], Sun et al. employed a combination of multilevel spectral features and multiview features for seizure detection. They utilized particle swarm optimization (PSO) for feature selection and support vector machine (SVM) as the classification algorithm to enhance the accuracy of identifying epileptic seizures. However, these approaches may ignore the fact that EEGs utilize a highly non-stationary signal and are extremely variable for intra- and inter-class. Actually, for these reasons, recent studies have turned to approaches based on deep learning.

Turner et al. applied deep belief network (DBN) to epilepsy detection, ignoring spatial information and time correlation [22]. Other works have shown similar drawbacks, such as with auto-encoder (AE) [23]. Khan et al. proposed an improved CNN architecture for seizure detection, using wavelet transform to construct the seizure features [24]. Ozcan et al. provided a 3D CNN utilizing FFT for feature transformation to accomplish the seizure prediction task [25]. This work, assisted by CNN, significantly promoted the ability to learn the spatial information of EEGs. Bashivan et al. provided an approach to transform the EEG signal into an EEG spectrum image and used them to predict the workload of the brain [26]. Based on this paradigm, the most representative work employed a hybrid network (convolutional neural network–long short-term memory units (LSTM), referred as CNN-LSTM) to automatically detect and predict epilepsy [27]. This hybrid model captured the spatial correlation of the EGG by the CNN model, in addition to considering the time correlation of the EEG through the LSTM model. As we know, LSTM is hard to train, displaying gradient explosion and gradient vanish, which result in a worse performance. From comprehensive consideration, the hybrid model may still have the drawback that it is difficult to obtain a balance between the two models. Ibrahim et al. introduced a patient-tailored method for EEG channel selection and seizure prediction, where PDFs of various signal attributes are analyzed to select bins for seizure prediction, followed by a decision fusion process for final classification [28]. Tang et al. proposed a multiview CGRN using fractal spectrum and PLV modularity for seizure feature extraction [29]. Li et al. further proposed fully convolutional nested LSTM, paying attention to high-level features for promoting the ability for seizure diagnosis [30]. Muhammad et al. proposed a three-step approach involving preprocessing scalp EEG signals with an advanced pipeline, extracting a combination of handcrafted and CNN-based features, and using LSTM for classification to predict seizures with high accuracy [31]. Hu et al. explored the applicability of transfer learning for deep learning models in epilepsy prediction, proposing a novel hybrid Transformer-based algorithm [32]. Deng et al. introduced a novel hybrid visual Transformer (HViT) framework, integrating CNN to bolster the ability to process local features [33]. Yuan et al. proposed a new hybrid deep learning framework that combines DenseNet and ViT through an attention fusion layer for seizure prediction. This architecture leverages DenseNet’s ability to capture hierarchical features and utilize parameters efficiently while also benefiting from self-attention mechanisms for global feature representation [34]. The results demonstrate the superiority of the Transformer-based model over pure CNN-based structures.

Despite their significant achievements, these works are based on an unmixed discrimination model, which suffers from lack of ability in modeling higher-order and hierarchical features of EEG signals, leading to poor capacity in recognizing and classifying cross-patient EEG datasets that are highly nonlinear and complex. In this paper, we attempt to propose a novel deep probability model assisted by self-attention and further optimized by dual-task learning for EEG-based seizure diagnosis and prediction. In detail, we introduce spike and slab units into the CRBM module for the purpose of capturing the average and covariance information of EEG data, further promoting the capacity in learning hierarchical and abstract representations. Meanwhile, the model utilizes a self-attention mechanism to dynamically weigh the importance of different time points in the EEG signals, thereby facilitating a more nuanced understanding of the temporal features.

## 3. Methodology

### 3.1. Database Description and Preprocessing

The largest publicly and freely accessible dataset for epilepsy prediction and detection tasks is the Children’s Hospital of Boston-Massachusetts Institute of Technology (CHB-MIT) dataset [35]. This dataset has been extensively explored in various studies for fair comparisons due to its comprehensiveness. It comprises data from 23 patients, including 5 males and 18 females who participated in the data collection task, distributed across 24 cases. Furthermore, the majority of the EEG data were recorded through 23 channels at a sampling frequency of 256 Hz, encompassing a total of 969 h of recordings with 198 seizures.

EEG signals comprise multiple sequential series corresponding to 23 electrodes (or 24 or 26 in some cases) positioned around the scalp, indicating that EEG data exhibit a high-dimensional distribution with spatial information. In this study, we utilized a subset of the CHB-MIT dataset, which includes 23 selected channels from 10 patients. We specifically classified the EEG signals into three categories: preictal, ictal, and interictal conditions. The preictal category encompasses the 60 min of EEG recordings immediately preceding the ictal period, whereas the ictal category consists of the ictal period itself. Furthermore, the remaining recordings were categorized as the interictal category. Based on these three categories, we constructed two distinct datasets for epilepsy prediction and detection, respectively.

To construct DatabaseI for epilepsy prediction, we focused on the preictal and interictal categories, excluding the ictal category. By distinguishing the preictal condition from the interictal condition, we aimed to accurately predict the onset of seizures. We selected 30 seizures from 10 patients for this task. For each patient, we used three preictal segments, totaling 180 min. The reason for choosing an equal amount of recording time from each patient was to maintain balance of intra- and inter-data. Additionally, we similarly selected 180 min of interictal data from each patient. This allowed us to perform a binary classification task to assess the sensitivity of our model for seizure prediction.

**Figure 1:**
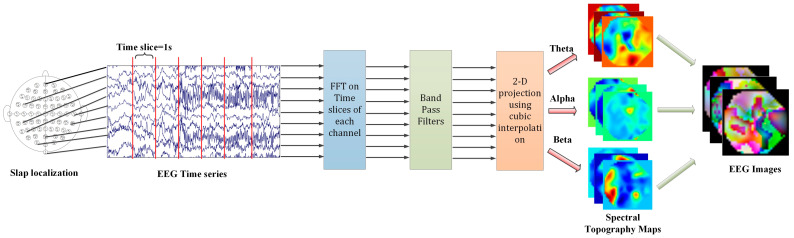
Overall flow graph of preprocessing.

Lastly, normalization and zero-component analysis (ZCA) whitening were applied to the processed datasets. An overall flowchart of the preprocessing steps is clearly illustrated in Figure 1.

In the preprocessing stage, we conducted thorough processing of the EEG signals from the CHB-MIT dataset to ensure data accuracy and the effectiveness of subsequent model training. The specific steps are as follows:

Firstly, we segmented the raw EEG signals into 2-second intervals. This time step was chosen based on the characteristics of EEG signals and analysis requirements, aiming to balance time resolution and frequency resolution. Next, we applied the fast Fourier-transform (FFT) to each 2-second segment of the EEG signals. FFT is an algorithm that converts time-domain signals into frequency-domain signals, allowing us to analyze the energy distribution of signals across different frequencies. Subsequently, we applied a low-pass filter to the frequency-domain data to remove high-frequency noise and retain the frequency ranges of interest. In this step, we treated each EEG channel as an independent measurement and filtered them separately. Based on the physiological characteristics of EEG signals and research purposes, we selected three specific frequency bands for further analysis: theta (4–7 Hz), which is associated with deep relaxation and meditation states; alpha (8–13 Hz), which is related to relaxation and awake states; and beta (13–30 Hz), which is linked to concentration and cognitive activities. The selection of these frequency bands was based on their known functions in brain activity and potential associations with neurological disorders such as epilepsy.

To convert the EEG spectrograms into a 2D image format suitable for model input, we employed cubic interpolation to resize the spectrograms to a resolution of 64 × 64. Cubic interpolation is a commonly used image scaling method that achieves smooth transitions while preserving image details. The EEG time series is segmented into 2-second intervals for each trial, resulting in 10,800 EEG spectral images with a 64 × 64 resolution for each patient. The entire dataset comprises 108,000 images (10,800 images per patient x 10 patients). Finally, we applied normalization and zero-component analysis (ZCA) whitening to the processed datasets. Normalization aimed to eliminate the dimensional differences between different features, making model training more stable. Figure 1 illustrates the overall flowchart of the preprocessing steps, including the specific operations and sequence of each step.

Furthermore, we constructed DatabaseII specifically for epilepsy detection. In this section, we focused on the preictal, ictal, and interictal categories. Given the particularly short duration of ictal periods compared to the other categories in the dataset, we carefully extracted 30 min of EEG recordings during the ictal phase for each of the 10 patients to form the ictal category. To ensure data balance across all categories, we also selected 30 min of interictal recordings. Additionally, we divided the 60-minute preictal condition into two sequential, non-overlapping fragments named PreI and PreII, each lasting 30 min. This segmentation aimed to capture the dynamic changes in EEG signals leading up to a seizure. However, it is important to note that distinguishing between PreI and PreII can be challenging due to the subtle differences in EEG patterns during these periods. This difficulty often results in high error rates when attempting to classify these two stages. The specific limitations in the segmentation process may include the overlap of EEG features between PreI and PreII as well as the variability in EEG patterns across different patients.

The specific preprocessing steps for DatabaseII were similar to those used for DatabaseI, with the exception that we chose a time step of 0.2 s for performing FFT on the EEG data. This shorter time step enabled us to generate more data samples, given the significantly smaller amount of EEG recordings in this dataset compared to DatabaseI. Finally, we constructed a four-class classification task with 9000 images in each category to demonstrate the performance of our method in seizure detection.

### 3.2. Overall Framework

**Figure 2:**
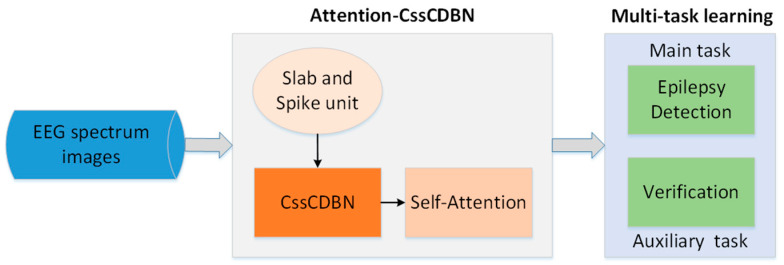
Overall framework of spatial-temporal EEGNet for epilepsy prediction and detection.

To investigate the spatio-temporal context within EEG spectrum images for epilepsy prediction and detection, we developed an innovative model termed spatial-temporal EEGNet, and the comprehensive framework is illustrated in Figure 2. As the foundational network within spatial-temporal EEGNet, attention-CssCDBN plays a pivotal role in joint feature extraction and classification through a sophisticated dual-task learning approach. The attention-CssCDBN architecture integrates a self-attention mechanism with a CssCDBN backbone, augmented by a dual-task learning paradigm. The self-attention module is designed to capture long-range dependencies and emphasize relevant features within the EEG signals, which are crucial for accurate epilepsy prediction and detection. This mechanism operates by assigning weights to different parts of the input sequence, allowing the model to focus on the most informative segments of the EEG data.

The dual-task learning framework comprises two intertwined tasks: the primary classification task of epilepsy EEG and an auxiliary verification task. The main task aims to classify EEG signals into epileptic or non-epileptic categories, leveraging the rich spatio-temporal features extracted by the network. The auxiliary task, inspired by face image verification techniques [14], serves as a regularization mechanism. It involves verifying the consistency of features extracted from different segments of the same EEG signal, promoting the learning of robust and generalizable representations.

The interaction between the self-attention module and the dual-task learning framework is crucial for the performance of model. The self-attention mechanism enhances the ability to attend to critical features, while the dual-task learning ensures that these features are both discriminative for classification and consistent across different parts of the signal. This interaction helps to mitigate overfitting, a common challenge when training deep models on limited EEG data.

In this section, we provide concise definitions for the aforementioned tasks. The training set V consists of pairs of images $(v_{1,i},v_{2,i})\in V$, each accompanied by a verification label $y_{ver,i}\in \left\{0,1\right\}$ that indicates whether the two samples belong to the same class (intra-class) or different classes (inter-class). We denote the diagnosis label of the subjects using the symbol $y_{main,i}\in \left\{0,1\right\}$. For the purpose of inference, appropriate adjustments are necessary. The cost function is defined in Equation (1), where the symbol $c_{ver}$ serves as a weighting factor to balance the significance between the auxiliary task and the main task. The structure of each component of the loss function is illustrated as follows.

(1) $$Loss=\sum_{(v_{1,i},v_{2,i})\in V}\left\{[loss_{main}(v_{1,i},y_{main,(1,i)})+loss_{main}(v_{2,i},y_{main,(2,i)})]+c_{ver}loss_{ver}(v_{1,i},v_{2,i},y_{ver,i})\right\}$$

Furthermore, we provide detailed descriptions of each task as follows:

(1) Main Task: Classification of Epilepsy EEG

We utilized EEG spectral images v∈V derived from individual subjects as input to train attention-CssCDBN for the purpose of accurately classifying each subject into their respective true class. The CssCDBN model was optimized through backpropagation following a pretraining phase, enabling it to minimize the classification error effectively. The main loss function is formulated as follows:

(2) $$loss_{main}=\frac{1}{N}\sum_{i=1}^{N}-y_{main,i}\log\;\hat{y_{main,i}}-(1-y_{main,i})\log\;(1-\hat{y_{main,i}})$$

(2) Auxiliary Task: Verification

Given a pair of EEG images $(v_{1},v_{2})\in V$ and corresponding label $y_{ver}\in \left\{0,1\right\}$, we utilized CssCDBN to determine whether two images originated from the same individual. Siamese CssCDBN (illustrated in Figure 2) was utilized to conduct identity verification, which consists of two identical sub-networks with shared weights. It is noteworthy that CssCDBN is treated as a discriminative special forward CNN. This approach alleviates the computational load through a weak approximation of variational learning and subsequently performs normalization. We can treat CssCDBN as the feature extractor function denoted as F(v). The sub-model F(v) with parameters w would produce features $F(v_{1};w)$ and $F(v_{2};w)$ while feeding in EEG images. The obtained features are low-dimensional and belong to the respective input images. The calculation formula for the Euclidean distance between output features is shown in Equation (2).

We consider the CssCDBN as a feature extractor function denoted as F(v). When EEG images are input into the sub-model F(v), it produces feature vectors *F*(*v*_1_;*w*) and *F*(*v*_2_;*w*). These feature vectors are low-dimensional representations of the input images. The Euclidean distance between the output features is calculated using the formula provided in Equation (3).

(3) $$D(v_{1},v_{2};w)={\left\|F(v_{1};w)-F(v_{2};w)\right\|}_{2}$$

To train our model for the verification task, we employed the Euclidean distance between the EEG features belonging to each image. For a given pair of EEG images $\left(v_{1},v_{2}\right)$, we define a cost function $loss_{ver}$ based on the estimation of whether the samples belong to the same identity. The cost functions for the identification and verification tasks are described in detail below, and we subsequently combine them to formulate the overall cost function. If $v_{1}$ and $v_{2}$ share the same identity, the cost function will be calculated as in Equation (4).

(4) $$loss_{vfs}(v_{1},v_{2};w)=\frac{1}{2}D{\left(v_{1},v_{2};w\right)}^{2}$$

For intra-class samples, the cost function tends to increase as the Euclidean distance between the features grows larger. Conversely, the cost function approaches zero if the features belong to the same identity. Additionally, when $v_{1}$ and $v_{2}$ are derived from different identities, the cost function can be computed using Equation (5).

(5) $$loss_{vfd}(v_{1},v_{2};w)=\frac{1}{2}{\left(\max(0,\delta -D(v_{1},v_{2};w))\right)}^{2}$$

As the Euclidean distance between the learning features decreases, the value of the cost function tends to increase. The parameter δ present in Equation (5) is referred to as the margin. The loss function is set to zero when the Euclidean distance between the features of two samples is significantly greater than the value of *δ*. Therefore, the margin δ allows our model to handle cases where samples from different identities generate similar features, thereby enhancing the model ability to better discriminate between inter- and intra-class images.

Furthermore, we combine the established cost functions $loss_{vfs}$ and $loss_{vfd}$ into a unified cost function $loss_{ver}$ to handle both cases. Given a label $y_{ver}\in \left\{0,1\right\}$, where $y_{ver}=1$ indicates that two samples belong to the same category and $y_{ver}=0$ indicates that they belong to different categories, the overall cost function is defined as in Equation (6).

(6) $$loss_{ver}=y_{ver}loss_{vfs}(v_{1},v_{2};w)+(1-y_{ver})loss_{vfd}(v_{1},v_{2};w)$$

As previously discussed, we trained our CssCDBN using an approach with stacked CssRBMs with forward-backward learning and backpropagation, which is both easy to infer and highly generalizable. Table 1 outlines the training process for the entire framework.

### 3.3. Attention-CssCDBN Descriptions

**Figure 3:**
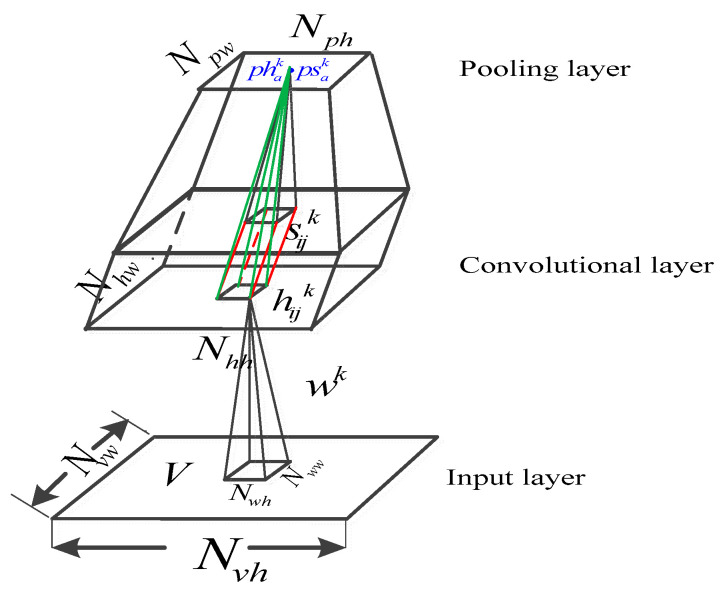
Detailed diagram of CssCRBM.

The main drawbacks of CRBM can be found in our previous work [15]. Here, we directly presented the energy function of the CssCRBM, which is illustrated in Equation (7). A detailed graph of the CssCRBM is shown in Figure 3.

(7) $$E\left(v,s,h\right)=-\overset{L}{\underset{l=1}{\sum }}\overset{K}{\underset{k=1}{\sum }}\overset{N_{hh,}N_{hw}}{\underset{i=1,j=1}{\sum }}{\left(v^{l}\Theta w^{l,k}\right)}_{ij}h_{ij}^{k}s_{ij}^{k}-\overset{K}{\underset{k=1}{\sum }}b^{k}\overset{N_{h,}N_{w}}{\underset{i=1,j=1}{\sum }}h_{ij}^{k}+\overset{L}{\underset{l=1}{\sum }}\overset{N_{vh},N_{vw}}{\underset{m=1,n=1}{\sum }}\frac{1}{2\sigma^{2}}{\left(v_{m,n}^{l}-c_{l}\right)}^{2}+\frac{1}{2}\overset{K}{\underset{k=1}{\sum }}\overset{N_{hh,}N_{hw}}{\underset{i=1,j=1}{\sum }}s_{ij}^{k}a_{ij}^{k}s_{ij}^{k}\;\;$$

where $v^{l},h_{ij}^{k}$ and $s_{ij}^{k}$ represent observations in the l−th channels, while there is a hidden spike and slab unit at coordinate (i,j) of the k−th feature map, respectively. $a_{ij}^{k}$ is a penalty term that imposes a large value on the slab unit $s_{ij}^{k}$ in the k−th feature map. K and L respectively indicate the number of convolutional kernels and the number of channels in observations (RGB image, L=3; Gray image, L=1). $w_{ij}^{l,k},c_{l},b^{k}$ are the weight value atcoordinate (i,j) of the k−th kernels in the l−th channel, the bias of visible units in the l−th channel, and the bias of spike units in the k−th feature map, respectively. Θ denotes convolutional operation.

**Figure 4:**
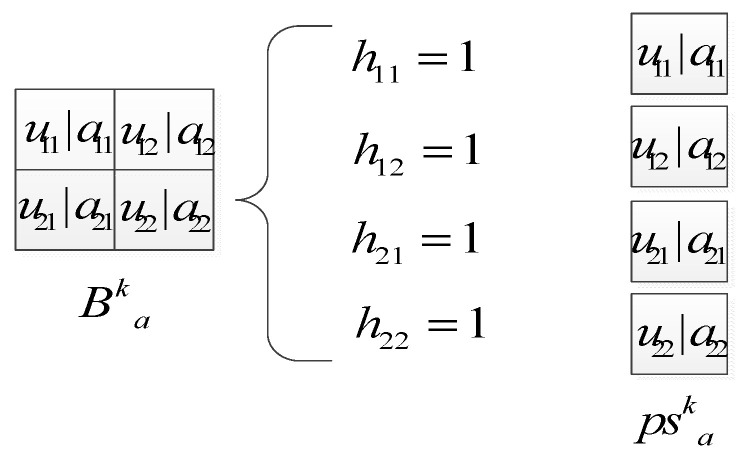
Interpretation of the slab-pooling.

The interpretation of the slab-pooling is illustrated in Figure 4. The states of the neurons in visible layer, spike units, and slab units are conditionally independent. The inferences of conditional probability function for each unit can be inferred as follows (without pooling) [14].

(8) $$Inference\;I:\;p(h_{ij}^{k}=1|v)=\sigma (\frac{1}{2a_{ij}^{k}}\overset{L}{\underset{l=1}{\sum }}{{\left(v^{l}\Theta w^{l,k}\right)}_{ij}}^{2}+b^{k})$$

(9) $$Inference\;II:\;p(s_{ij}^{k}|v,h)=\mathbb{N}(\frac{1}{a_{ij}^{k}}\overset{L}{\underset{l=1}{\sum }}{\left(v^{l}\Theta w^{l,k}\right)}_{ij}h_{ij}^{k},\frac{1}{a_{ij}^{k}})$$

(10) $$Inference\;III:p(v_{mn}^{l}|s,h)=\mathbb{N}(\sigma^{2}\sum_{i,j=1}^{N_{hh},N_{hw}}\overset{K}{\underset{k=1}{\sum }}(h_{ij}^{k}s_{ij}^{k}\Theta \tilde{w^{l,k}}+c_{l}),\sigma^{2})$$

Furthermore, we introduced a novel operation termed dual-variable probabilistic max-pooling, which is similar to the probabilistic max-pooling employed in CRBMs. Our improvement effectively amalgamates the covariance information originating from both top-down and bottom-up layers by means of stacking multiple CssCRBMs. In this context, we present the energy function for the CssCRBM augmented with dual-variable probabilistic max-pooling. The detailed formula is defined as follows [14].

(11) $$\begin{array}{c} E\left(v,s,h\right)=-\overset{L}{\underset{l=1}{\sum }}\overset{K}{\underset{k=1}{\sum }}\overset{N_{hh,}N_{hw}}{\underset{i=1,j=1}{\sum }}(v^{l}\Theta w^{l,k}{)}_{ij}h_{ij}^{k}s_{ij}^{k}-\overset{K}{\underset{k=1}{\sum }}b^{k}\overset{N_{h,}N_{w}}{\underset{i=1,j=1}{\sum }}h_{ij}^{k}+\overset{L}{\underset{l=1}{\sum }}\overset{N_{vh},N_{vw}}{\underset{m=1,n=1}{\sum }}\frac{1}{2\sigma^{2}}(v_{m,n}^{l}-c_{l}{)}^{2}+\frac{1}{2}\overset{K}{\underset{k=1}{\sum }}\overset{N_{hh,}N_{hw}}{\underset{i=1,j=1}{\sum }}s_{ij}^{k}a_{ij}^{k}s_{ij}^{k}\\ \;\;\;subject\;to:\;\sum_{(i,j)\in B_{a}}h_{ij}^{k}\leq 1,\forall \;k,a \end{array}$$

It is noteworthy that the CssCRBM incorporates two sets of pooling units, denoted as $\left\{ps_{a}^{k}\right\}$ and $\left\{ph_{a}^{k}\right\}$. Equation (11) reveals several constraints imposed on the spike hidden units: at most one unit may be active within each block, and a pooling unit $\left\{ph_{a}^{k}\right\}$ is activated while a corresponding detection unit is active. This behavior is analogous to probabilistic max-pooling. Additionally, the slab pooling units function similarly to maximum mean detection units. Lastly, an element-wise multiplication operation is conducted between $\left\{ps_{a}^{k}\right\}$ and $\left\{ph_{a}^{k}\right\}$. The inference of the conditional probability function, facilitated by the dual variable probabilistic max-pooling, is delineated as follows.

(12) $$\mathrm{Inference}\;I:\;p(h_{ij}^{k}=1|v)=\frac{\exp(\frac{1}{2a_{ij}^{k}}\overset{L}{\underset{l=1}{\sum }}{{\left(v^{l}\Theta w^{l,k}\right)}_{ij}}^{2}+b^{k})}{1+\sum_{(i^{\prime },j^{\prime })\in B_{a}}\exp(\frac{1}{2a_{i^{\prime }j^{\prime }}^{k}}\overset{L}{\underset{l=1}{\sum }}{{\left(v^{l}\Theta w^{l,k}\right)}_{i^{\prime }j^{\prime }}}^{2}+b^{k})}$$

(13) $$p(ph_{a}^{k}=0|v)=\frac{1}{1+\sum_{(i^{\prime },j^{\prime })\in B_{a}}\exp(\frac{1}{2a_{i^{\prime }j^{\prime }}^{k}}\overset{L}{\underset{l=1}{\sum }}{{\left(v^{l}\Theta w^{l,k}\right)}_{i^{\prime }j^{\prime }}}^{2}+b^{k})}$$

(14) $$\mathrm{Inference}\;\mathrm{II}:\;p(s_{ij}^{k}|v,h)=\mathbb{N}(\frac{1}{a_{ij}^{k}}\overset{L}{\underset{l=1}{\sum }}{\left(v^{l}\Theta w^{l,k}\right)}_{ij}h_{ij}^{k},\frac{1}{a_{ij}^{k}})$$

(15) $$p(ps_{a}^{k}|v)=N(\underset{(i,j)\in B_{a}}{\max}\frac{1}{a_{ij}^{k}}\overset{L}{\underset{l=1}{\sum }}{\left(v^{l}\Theta w^{l,k}\right)}_{ij}h_{ij}^{k},\frac{1}{a_{ij}^{k}})$$

Dual-variable probabilistic max-pooling is defined in Equation (16).

(16) $$\bar{p(ph_{a}^{k}=1|v)}\;\bar{p(ps_{a}^{k}|v)}$$

Here, $\bar{p(\cdot )}$ denotes a sample from p(·). 

The training process of the CssCRBM model is carefully depicted in Table 2.

Going Deeper for Epilepsy Detection: Motivated by the construction of CDBN with RBMs, we extended the CssCRBM to a novel deep generative model named CssCDBN for better learning of hierarchical representations. In detail, we trained the bottom CssCRBM following the procedure in Table 2 to achieve better initialization. Subsequently, we employed the hybrid feature maps from the preceding CssCRBM as input for the next CssCRBM, facilitating the straightforward scaling of the model depth. The aforementioned operations considerably improved the feature extraction capabilities within the receptive field, further contributing to the learning of hierarchical and abstract representations. The configuration of the CssCDBN module proposed in this paper is depicted in Table 3.

Subsequently, we incorporated an attention module to selectively emphasize the contextual temporal features embedded within EEG signals. It can be regarded as a Transformer encoder block, comprising a self-attention (SA) block and a Two-Layer Perceptron block. Both blocks are interconnected through residual connections, a strategy that has been empirically proven to bolster the overall performance and training stability of deep neural networks.

**Figure 5:**
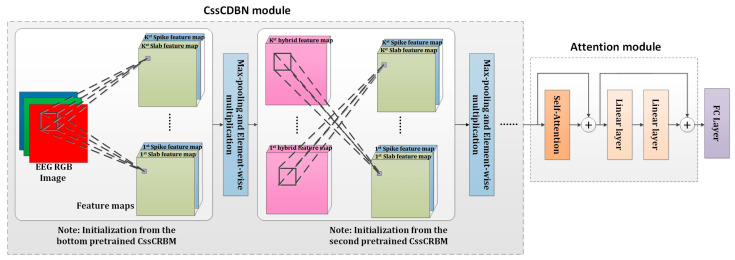
Overall structure of attention-CssCDBN.

As the core part of the attention module, the SA block provides an effective approach in capturing the comprehensive local and global context within EEG images. By leveraging sophisticated representations for Query (Q), Key (K), and Value (V), SA facilitates a nuanced understanding of the spatial and temporal dependencies inherent in EEG data. This approach transcends traditional methods that rely heavily on the softmax function for attention weight normalization, as SA inherently encodes the relevance of each feature without the need for such additional operations. The integration of the Two-Layer Perceptron block further augments the SA block’s capabilities by introducing non-linearity and enabling the model to learn more complex patterns and relationships within the EEG signals. The residual connections, which add the output of each block to its input, help mitigate the vanishing gradient problem and facilitate the propagation of gradients through deeper layers, thus enhancing the model’s ability to learn from the EEG data. Figure 5 depicts the comprehensive architecture of the attention-CssCDBN, referred to as the spatial-temporal EEGNet.

Once obtaining the pretrained model, we can treat CssCDBN as a special CNN. Different from conventional CNN, our CssCDBN utilizes two classes of feature maps that are binary and real-valued, respectively. For better training, we used several techniques during the fine-tuning stage. Firstly, we used Equation (17) to replace Equation (8) in order to extract spike feature maps. Secondly, we used Equation (18) to replace Equation (9) in order to extract slab feature maps. Finally, we used $h_{ij}^{k}s_{ij}^{k}$ to obtain hybrid feature maps instead of utilizing the sampling method, as in Equation (16).

(17) $$h_{ij}^{k}=relu\;(\frac{1}{2a_{ij}^{k}}\overset{L}{\underset{l=1}{\sum }}{{\left(v^{l}\Theta w^{l,k}\right)}_{ij}}^{2}+b^{k})$$

(18) $$s_{ij}^{k}=\frac{1}{a_{ij}^{k}}\overset{L}{\underset{l=1}{\sum }}{\left(v^{l}\Theta w^{l,k}\right)}_{ij}h_{ij}^{k}+\frac{1}{a_{ij}^{k}}randn(0,1)$$

## 4. Results

To evaluate the specific advantage of the proposed method, we conducted experiments on the preprocessed dataset obtained from CHB-MIT. For our dataset, we employed 10-fold cross-validation. We conducted several experiments that can be distributed into three categories: (1) competition results and analysis; (2) parameter sensitivity analysis of dual-task learning; and (3) visualization analysis. All the experiments were conducted on desktop with a Ubuntu 18.04, 64-bit system. We utilized two GTX2080 GPUs for computation accelerating with deep learning libraries named Tensorflow.

### 4.1. Spatial-Temporal EEGNet and Competitive Methods

#### 4.1.1. Spatial-Temporal EEGNet

Since our spatial-temporal EEGNet has more parameters compared to the quantity of the existing data, it is pivotal to utilize an appropriate regularization method in the classification task.

The specific parameter setting of the model training was as follows. We mainly conducted parameter setting during the fine-tuning stage and classification process. The spatial-temporal EEGNet was finely tuned by the approach depicted in Table 2. We set the initial learning rates {λ, η} as 0.01. For the verification of spatial-temporal EEGNet’s expectation, the batch size was set to 20. CD algorithm was adopted to train all the CssCRBM layers, and the step was set to 5. We adopted Adam as the optimizer for training. The coefficients of the contractive penalty term from 1st layer to 4th layer were set to 0.1, 0.1, 0.2, and 0.2. The coefficients of auxiliary tasks {c*_ide_*, c*_ver_*} were set to 0.07 and 0.11, respectively. Equations (8)–(16) specifically give the calculation of approximate inferences after 100 iterations.

#### 4.1.2. Competitive Methods

In this study, we established a series of models to serve as pertinent baseline methods and evaluated their performance in the task of seizure detection. Furthermore, we referenced several baseline and state-of-the-art algorithms, conducting comparative experiments between our proposed spatial-temporal EEGNet and these aforementioned methods. The detailed framework and network parameters of the baseline methods are depicted as follows.

DBN-3: We stacked three RBMs for the construction of a specific DBN. In detail, we utilized Gaussian-binary RBM as the first layer, which is of great efficiency in extracting the continuous information existing in the EEG data. We further adopted two binary RBMs for the last two layers. Each layer has 512, 512, and 128 neurons from bottom to top, respectively. The structure of DBN-3 is the same as that in [36].

GDBM-2: GDBM is an extension of DBM. We adopted a GDBM-2 architecture in this study, while the hyper parameter was kept consistent with that in [37].

VGG-8: The employed VGG for our validation experiment is diverse from the original version [38] since the training would be faced with overfitting. The configurations of VGG-8 are illustrated in Table 4.

GoogleNet: The original version of GoogleNet [8] was unsuitable for our task due to its huge network parameters. Here, we adopted two inception modules of GoogleNet V2, which contains a 2 × 2 max-pooling and three diverse convolutional kernels with the sizes of 1 × 1, 3 × 3, and 5 × 5, assisted by batch normalization. A 2 × 2 average-pooling layer was concatenated to each inception module.

ResNet-10: ResNet has been demonstrated to be an efficient network in the task of image classification [9]. Here, we adopted a specific structure with three basic blocks. Each block consists of 3 × 3 convolutions, 3 × 3 convolutions, and 1 × 1 convolutions, from shallow to deep.

CDBN: A four-layer CDBN [6] was adopted for our task with sparse constraint (*p* = 0.001). The architecture of CDBN was kept consistent with our model as described in Table 3.

Vision Transformer (ViT) [39]: The Transformer encoder consists of 12 stacked layers, with the number of attention heads set to 16 within each layer. Each attention layer is followed by a position-wise fully connected feedforward network, with a hidden layer dimension that is four times the embedding dimension.

### 4.2. Epilepsy Prediction on DatabaseI

In this section, we assess the specific performance of our proposed model using DatabaseI by conducting binary classification tasks to evaluate its sensitivity in predicting seizures. Sensitivity and false-positive rate (FPR) were employed as the evaluation metrics. Sensitivity measures the ratio of true-positive predictions among all actual positive instances, while FPR indicates the ratio of false-positive predictions among all actual negative instances. High sensitivity and low FPR directly reflect significant performance capacity for the detection of real ictal and non-pathogenic features. Sensitivity and FPR are calculated as Equations (19) and (20), respectively.

(19) $$Sensitivity=\frac{TP}{TP+FN}$$

(20) $$FPR=\frac{FP}{TN+FP}$$

where *TP* and *FP* indicate true and false positive, while *TN* and *FN* represent true and false negative, respectively.

Samples from the preictal and interictal periods were designated as positive and negative, respectively. An epilepsy onset was considered accurately predicted when at least one sample within the preictal category was correctly identified as positive. Thus, we can calculate the prediction time of an epilepsy onset by the occurrence of the first positive sample. This prediction time serves as a crucial indicator for assessing the early detection capabilities of our model. Competitive experiments were conducted to compare our EEGNet with a range of baseline models, including DBN-3, GDBM-2, VGG-8, GoogleNet, ResNet-10, CDBN, and ViT. The configurations of both our model and the competitive models are detailed in Section 4.2.

The sensitivity, FPR, and prediction time for the classification task are presented in Table 5, Table 6, and Table 7, respectively. These results reveal that our spatial-temporal EEGNet achieved a sensitivity of 98.5%, an FPR of 0.041/h, and a prediction time of 50.92 min, demonstrating significant advantages over the baseline methods. Specifically, CDBN outperformed DBN-3 and GDBM-2, indicating that the combination of a convolutional mechanism with a DBN is more effective in learning spatial representations than traditional probabilistic graphical models. Our spatial-temporal EEGNet significantly surpassed CDBN, illustrating that the series of improvements and strategic applications we implemented based on CDBN significantly enhanced the model’s performance. Additionally, our ssEEGNet outperformed standard CNNs, including VGG-8, GoogleNet, and ResNet-10, primarily due to its more powerful feature extraction capabilities and the assistance provided by the dual-task learning framework. This reveals that our proposed model exhibits superior performance compared to ViT. These results not only emphasize the potent efficacy of the several strategies integrated into our model but also reveal the inherent challenges ViT faces in effectively processing temporal features. Additionally, the observation that ViT surpassed all other baseline methodologies underscores the pivotal significance of incorporating attention mechanisms in feature extraction from EEG images.

It is noteworthy that our method achieved the best sensitivity and FPR across all 10 subjects, indicating its robust performance against inter- and intra-class variations. Furthermore, our method achieved the best prediction time for 8 out of the 10 subjects, with exceptions for subjects 4 and 9. This suggests that seizure prediction time may exhibit some stochasticity due to cross-patient variations, although our spatial-temporal EEGNet achieved superior performance in average indices.

Table 8 gives the comparison of our spatial-temporal EEGNet with several state-of-art methods. All comparison methods were experimentally evaluated on the CHB-MIT dataset, ensuring fairness and validity in the comparison.

The results reveal that our method has 4.0% higher sensitivity compared with the state-of-art method and 0.041% lower FPR than another current method. Further, our spatial-temporal EEGNet achieved a better prediction time by 12.57 min compared to the state-of-art method. This indicates that our method performed better in learning representations from EEG-based seizure features, further demonstrating its significant efficiency in discriminating different features and its generalization ability.

### 4.3. Epilepsy Detection on DatabaseII

#### 4.3.1. Classification Results

In this section, we perform a four-class classification task based on DatabaseII to verify the significant performance of our spatial-temporal EEGNet on seizure detection. Different from Section 4.2, we utilize accuracy as the assessment indicator. as it is more representative of the multi-category classification task. A high accuracy specifically reveals the significant performance of discriminating diverse states. Accuracy is calculated with Equation (21).

(21) $$Accuracy=\frac{TP+TN}{TP+FP+FN+TN}$$

The detailed segmentation of the preictal state holds profound significance, as the accurate detection of this state is crucial for the early prediction of epileptic seizures. This approach also serves as a valuable supplement to epilepsy prediction tasks. By enabling precise seizure detection, it allows for prompt intervention. However, this task presents a substantial challenge, primarily due to the features of PreI and PreII segmented from the interictal state, which exhibit minimal differences.

In our study, we conducted a comprehensive comparison between our proposed spatial-temporal EEGNet and several state-of-the-art models, including DBN-3, GDBM-2, VGG-8, GoogleNet, ResNet-10, CDBN, and ViT. Detailed configurations for all these models are meticulously outlined in Section 4.1. The results of this comparative analysis, presented in Table 9, reveal that our spatial-temporal EEGNet achieved an impressive accuracy of 94.1%, which is notably 3.3% higher than that attained by ViT, the best-performing algorithm among the comparison models. This substantial improvement highlights the superior capability of our method in discerning intricate features and capturing subtle variations across different stages of epileptic activity, namely the interictal, PreI, PreII, and ictal states.

We further analyzed the structural advantages and experimental evidence that contribute to the superiority of spatial-temporal EEGNet over the existing models. Specifically, spatial-temporal EEGNet, through its unique design, is able to more effectively fuse spatial and temporal information, which is crucial for processing electroencephalogram (EEG) signals with spatio-temporal characteristics. Its carefully crafted network architecture, including spatio-temporal convolutional layers, slab and spike operation, and attention mechanisms, enables the model to more accurately capture key features in EEG signals and distinguish between subtle differences among different stages of epileptic activity.

#### 4.3.2. Ablation Analysis

In this section, we conduct an ablation analysis to assess the impact of attention mechanism and dual-task learning, as they perform significant roles in promoting the final effect of our method. Table 10 shows how the attention strategy and verification task impact the consequences. It reveals that the baseline model (dual-task learning and attention absent) obtained an accuracy of 86.1%, which is lower than GoogleNet due to the lack of unlabeled data for pre-training. When the attention mechanism was integrated into the baseline model, a notable increase in accuracy was observed from 86.1% to 90.3%. This suggests that the attention mechanism is effective in enhancing model performance, possibly by facilitating better focus on crucial features and temporal sequences within the data. Furthermore, the addition of dual-task learning, in the absence of the attention mechanism, also led to an improvement in accuracy from 86.1% to 91.9%. This indicates that dual-task learning positively influences model performance, potentially through the simultaneous learning of multiple related tasks, further enhancing the generalization capability of model. When both the attention mechanism and dual-task learning were incorporated into the model, the highest accuracy was attained. This demonstrates a synergistic effect between these two components, jointly contributing to optimal model performance.

#### 4.3.3. Visualization Verification

**Figure 6:**
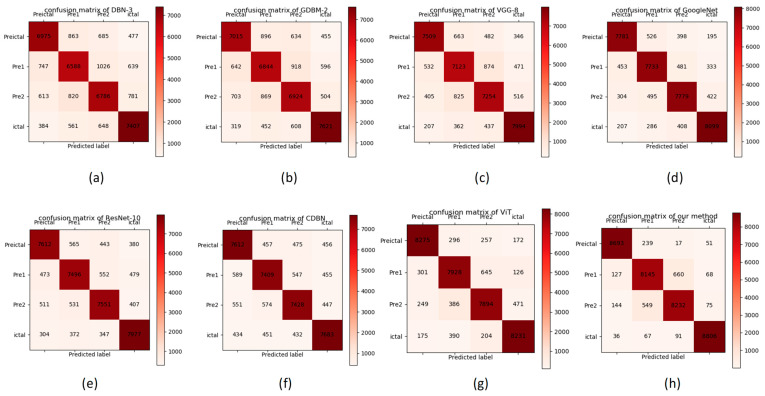
Confusion matrix: (**a**) DBN-3, (**b**) GDBM-2, (**c**) VGG-8, (**d**) GoogleNet, (**e**) ResNet-10, (**f**) CDBN, (**g**) ViT, and (**h**) our method.

In this section, we mainly focus on the discrimination between PreI and PreII for the purpose of accurately predicting the seizure ictal time. According to Figure 6, our ssEEGNet achieved the best performance in discriminating between PreI and PreII, showing that ssEEGNet could specifically distinguish different periods of the preictal state compared with the other models. We can also infer that there was still a high level of false identification between PreI and PreII compared with the other states in all the other models, including our ssEEGNet, due to the particular feature property.

In this section, we visualize the results by calculations for each method, respectively, as shown in Figure 6, to construct a more detailed comparison. DatabaseII contains four classes with 9000 EEG images existing in each class, according to Section 3.1. The confusion matrix presents the classification of samples in a matrix format, comparing the predictions of model with the actual labels. Here, we provide the confusion matrix through which we can infer that challenges exist in two aspects, mainly reflected in the indistinguishability of PreI and PreII, which are indistinguishable from adjacent states on the timeline. This phenomenon is mainly caused by the fact that the feature variations existing in adjacent states are inconspicuous, especially concerning PreI and PreII. In this context, our focus is on the accurate discrimination between PreI and PreII, which is crucial for predicting the seizure ictal time. According to Figure 6, it is evident that our proposed spatial-temporal EEGNet exhibits the best performance in discriminating between PreI and PreII, as indicated by the higher accuracy rates and lower misclassification rates between these two states compared to other models. This indicates that our method is capable of specifically distinguishing between different periods of the preictal state, outperforming the other models considered.

Furthermore, it is noteworthy that the false identification rate between PreI and PreII remains relatively high compared to other states across all models, including our method. This can be attributed to the inherent characteristics of the features, which make it challenging to differentiate between these two states. Nonetheless, the results demonstrate the efficacy of ssEEGNet in addressing this challenge, highlighting its potential for accurate seizure prediction.

**Figure 7:**
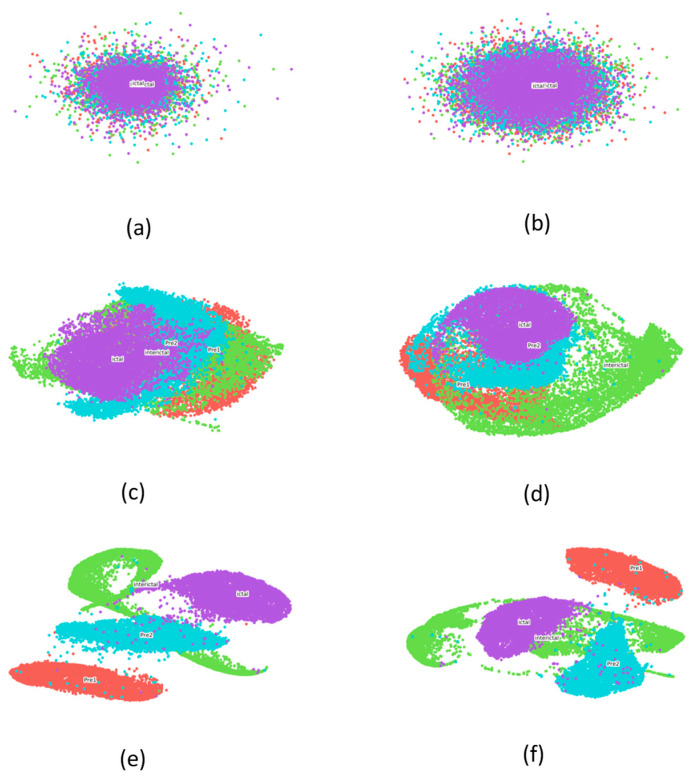
EEG representations visualization by t-SNE. (**a**) The two-dimensional map of original data. (**b**–**e**) The outputs of each hidden layer in CssCDBN. (**f**) The final output.

For further validation of our approach, we employed a dimensionality reduction algorithm to embed the high-dimensional representations into a two-dimensional space. The high-dimensional data were collected from the outputs of each hidden layer, allowing us to investigate the intrinsic peculiarities of the model through a layer-by-layer analysis. Given its superior efficiency in visualizing high-dimensional data compared to PCA, we utilized t-SNE for the visualization of each layer’s output. The embedding results are presented in Figure 7. Specifically, Figure 7a depicts the embedding result of the input data, providing a baseline for comparison. Figure 7b–e represent the outputs of each hidden layer in our model (CssCDBN), showcasing the progressive transformation of the data as they pass through the network. Figure 7f shows the final result obtained by the attention module, which refines the representations and enhances the discriminability between different states.

By comparing Figure 7f with the other figures, we can infer that our method is highly effective in epilepsy detection, achieved through the stacking of appropriate layers and the introduced novel strategy. The representations acquired by deeper layers are more abstract and discriminative compared to those from shallower layers. Notably, Figure 7c,d distinctly highlight the difficulty in differentiating between PreI and PreII states, a challenge we previously investigated. These findings further underscore the efficacy and challenges associated with our approach to epilepsy detection.

## 5. Discussion

In this study, we primarily conducted our tasks on two distinct databases for both prediction and detection purposes, which sets our work apart from that of other researchers. For DatabaseI, we focused on binary classification tasks to assess the sensitivity of our model in predicting seizures, thereby enabling more accurate prediction of epilepsy onset. This is particularly relevant for patients to be able to take early preventive measures.

For DatabaseII, we performed a four-class classification task to evaluate the performance in seizure detection, which posed a significant challenge due to the subtle variations among interictal, PreI, and PreII states. Despite the encountered difficulties, the task on DatabaseII allowed us to further investigate the detection capabilities of our proposed method in distinguishing between real ictal and non-pathogenic features. Previous studies have primarily relied on traditional machine learning algorithms or an unmixed discrimination model, leading to shortcomings in extracting higher-order and hierarchical features within EEG signals. However, our method, integrating probabilistic graphical models with attention mechanisms within a dual-task learning framework, showed superior performance in terms of accuracy and robustness. Furthermore, our method can deal with cross-patient EEG datasets that are highly nonlinear and complex.

The significant performance of our proposed method is primarily attributed to the integration of probabilistic graphical models with attention mechanisms, which are further embedded within a dual-task learning framework. Because our approach did not perfectly discriminate between PreI and PreII, this remains an area for future exploration. We suggest potential solutions, such as incorporating more sophisticated feature extraction techniques or utilizing machine learning algorithms that are better suited for handling subtle differences in data.

As in our previous work, CssCDBN demonstrated remarkable performance in image feature modeling. However, there is still potential to enhance its modeling ability by integrating a hierarchical representation learning strategy. In this study, we optimized the structure of CssCDBN by introducing an attention module and incorporating a dual-task learning framework that can be viewed as a soft constraint imposed on the networks. The auxiliary task significantly improved generalization and increased attention to intra- and inter-class variations. Through these two key innovations, we ultimately demonstrated the exceptional efficiency of our spatial-temporal EEGNet in learning representations from EEG images.

Although our method showed exceptional efficiency in learning representations from EEG images, it still experienced difficulties in dealing with the temporal features of EEG signals. We will conduct future research mainly on the integrating of CssCDBN with more efficient strategies for temporal context analysis. We look forward to building a more advanced probability-generative architecture base on this work that will focus more on temporal features, further exploring a better strategy to solve this problem.

## 6. Conclusions

In this paper, we introduce a novel deep discriminative probability model enhanced by an attention module and dual-task learning framework for EEG-based seizure prediction and detection. Our proposed spatial-temporal EEGNet comprises three key components: an enhanced discriminative deep convolutional generative network, an attention module, and an assisting dual-task learning framework, which collectively contribute to its superior performance. Compared to traditional probabilistic graphical models, our model exhibits greater efficiency due to the incorporation of slab and spike features within the convolutional layers, enabling the capture of higher-order and hierarchical features, while the attention module focuses more on temporal features. Furthermore, our work distinguishes itself from existing approaches through the adoption of verification tasks to mitigate overfitting. This strategy enhances the robustness and consistency of our model in addressing intra- and inter-class variations.

The experimental results demonstrate that our method outperforms the state-of-the-art and baseline methods in learning abstract representations from EEG images. These results highlight the promising potential of our spatial-temporal EEGNet for enhancing seizure detection and prediction in home-care systems.

## Figures and Tables

**Table 1:** Training process of overall framework.

◆ Given: a training dataset comprising *N* vectors ${\left\{v_{n},y_{n,main}\right\}}_{n=1}^{N}$ with hyper parameters {batchsize, epoch, η, c*_ver_*} ◆ Initialize trainable parameters θ from the pretrained models ◆ For i = 1 to epoch: ➢ For *j* = 1 to int (*N/batchsize*): v(0) = v[(*i* − 1) * *batchsize*: *i* * *batchsize*] $y_{main}\left(0\right)\;$ = $\;y_{main}$ [(*i* − 1) * *batchsize*: *i* * *batchsize*] Given v(0), $y_{main}\left(0\right)$, optimize cross entropy using back propagation and obtain optimized parameters $\nabla \theta_{main}$ Split v(0) into two groups equally and randomly combine two EEG images in order to produce training pairs; lastly, feed these training pairs into Siamese CssCDBN and use back propagation to obtain optimized parameters $\nabla \theta_{ver}$ Update weights using $\theta :\theta -\eta \left(\nabla \theta_{main}+c_{ver}*\nabla \theta_{ver}\right)$
End For End for

**Table 2:** The training process of the CssCRBM model.

◆ Given: a training set of N data vectors ${\left\{v_{n}\right\}}_{n=1}^{N}$ and setting hyper parameters ψ={batchsize,epoch,K,λ,η} ◆ Randomly initialize trainable parameters *θ* ◆ For *i* = 1 to *epoch*: ✧ For *j* = 1 to int (N/batchsize): v(0)=v[(i−1)·batchsize:i·batchsize] Sample h(0) and s(0) from Equations (12) and (14) For n = 1 to K: Sample v(n) from Equation (10), sample h(n) and s(n) from Equations (12) and (14) End For Update weights with samples from above $\theta (t+1)=\theta -\eta (\nabla \theta_{1}+\lambda \nabla \theta_{2})$
End For End for

**Table 3:** Configuration of CssCDBN module.

1st Layer	Input layer, size of input (batchsize,3,64,64)
	CssCRBM layer, size of kernels (36,3,3,3)
	Pooling layer, size of pooling (2,2)
2nd Layer	Input layer, size of input (batchsize,36,31,31)
	CssCRBM layer, size of kernels (64,36,3,3), padding = 1
	Pooling layer, size of pooling (2,2)
3rd Layer	Input layer, size of input (batchsize,64,15,15)
	CssCRBM layer, size of kernels (128,64,3,3), padding = 1
	Pooling layer, size of pooling (2,2)
4th Layer	Input layer, size of input (batchsize,128,7,7)
	CssCRBM layer, size of kernels (128,128,3,3), padding = 1
	Pooling layer, size of pooling (2,2)

**Table 4:** Configurations of VGG.

Layer	Conv3-32	Conv3-32	Conv3-32	Conv3-64	Conv3-64	Conv3-128	Conv3-128	FC-512
Kernel size	64 × 3 × 3	64 × 3 × 3	64 × 3 × 3	64 × 3 × 3	64 × 3 × 3	128 × 3 × 3	128 × 3 × 3	-
Convolution Stride	1	1	1	1	1	1	1	-
Max-pooling	-	-	2 × 2	-	2 × 2	-	2 × 2	
Pooling stride	-	-	2	-	2	-	2	-

**Table 5:** Classification sensitivity for DBN-3, GDBM-2, VGG-8, GoogleNet, ResNet-10, CDBN, ViT, and our method.

Subjects	Sensitivity %							
	DBN-3	GDBM-2	VGG-8	GoogleNet	ResNet-10	CDBN	ViT	Our EEGNet
1	73.1	77.2	82.9	88.5	86.4	79.3	91.4	96.5
2	82.3	85.8	93.8	92.1	90.6	85.2	95.1	98.9
3	88.7	89.2	92.6	95.5	93.1	92.0	96.5	99.8
4	84.5	86.4	92.4	97.2	96.3	88.2	94.2	98.4
5	89.2	91.3	97.1	98.7	96.5	91.6	96.3	99.7
6	77.4	75.6	81.8	86.1	83.0	83.1	91.1	97.3
7	80.5	81.7	85.0	90.3	86.3	87.3	92.2	98.2
8	81.4	85.4	87.1	89.4	86.1	84.8	94.3	99.0
9	86.3	89.0	94.5	87.3	84.9	88.2	94.6	99.5
10	75.6	74.4	79.8	84.9	80.8	76.3	91.3	97.7
Average	81.9	83.6	88.7	91.0	88.4	85.6	93.7	98.5

**Table 6:** Classification FPR for DBN-3, GDBM-2, VGG-8, GoogleNet, ResNet-10, CDBN, ViT, and our method.

Subjects	FPR/h							
	DBN-3	GDBM-2	VGG-8	GoogleNet	ResNet-10	CDBN	ViT	Our EEGNet
1	0.297	0.235	0.208	0.164	0.177	0.254	0.125	0.052
2	0.241	0.192	0.171	0.133	0.152	0.220	0.074	0.040
3	0.258	0.226	0.175	0.155	0.168	0.195	0.091	0.032
4	0.240	0.187	0.163	0.139	0.157	0.210	0.102	0.043
5	0.249	0.219	0.172	0.135	0.160	0.199	0.078	0.046
6	0.277	0.204	0.169	0.146	0.171	0.223	0.113	0.057
7	0.259	0.227	0.162	0.153	0.165	0.211	0.086	0.063
8	0.278	0.250	0.190	0.149	0.169	0.213	0.090	0.053
9	0.252	0.217	0.184	0.140	0.158	0.209	0.099	0.039
10	0.299	0.253	0.196	0.166	0.153	0.236	0.072	0.061
Average	0.265	0.221	0.179	0.148	0.163	0.217	0.093	0.041

**Table 7:** Prediction time for DBN-3, GDBM-2, VGG-8, GoogleNet, ResNet-10, CDBN, ViT, and our method.

Subjects	Prediction Time/Minutes							
	DBN-3	GDBM-2	VGG-8	GoogleNet	ResNet-10	CDBN	ViT	Our EEGNet
1	21.34	26.45	34.76	30.23	32.35	29.17	33.06	49.23
2	29.58	24.19	27.88	48.75	43.60	25.02	42.19	57.58
3	31.79	38.96	29.13	42.16	49.12	43.88	42.05	50.23
4	24.33	34.18	36.90	53.22	55.37	51.49	48.17	50.05
5	22.92	27.35	26.05	32.84	28.14	36.54	44.02	49.83
6	28.12	22.19	35.15	30.17	36.09	39.25	49.65	51.83
7	31.56	26.28	40.28	35.43	30.79	29.30	45.83	54.29
8	29.14	32.70	22.43	38.04	26.56	38.12	46.20	50.52
9	23.87	38.68	34.79	57.10	55.46	32.71	42.79	42.37
10	25.55	20.32	29.13	49.86	37.22	26.62	39.54	53.19
Average	26.82	29.13	31.65	41.78	39.47	35.21	43.35	50.92

**Table 8:** Comparison sensitivity, FPR, and prediction time for SVM, LDA, CNN, 3D CNN, multiview CGRN, CNN-LSTM, hybrid Transformer, and our method.

Method	Features	SEN (%)	FPR (/h)	Prediction Time (min)
SVM [19]	Phase locking value	82.44	-	-
LDA [20]	Common special pattern statistics	81	0.47	38.35
CNN [24]	Wavelet transform coefficients	87.8	0.147	5.83
3D CNN [25]	Spectral power, statistical moments, Hjorth parameters	87.01	0.186	-
Statistical framework [28]	Derivative, local variance, median filtering	90.3	-	22.63
Multi view CGRN [29]	Fractal spectrum, relative band energy, PLV modularity	94.5	0.118	27.15
CNN-LSTM [31]	Short time Fourier-transform	93.8	-	19.5
Hybrid Transformer [32]	Wavelet transform	91.7	0.00	-
HViT-DUL [33]	Data uncertainty learning	87.9	0.056	-
STFT + DenseNet–ViT [34]	Time-frequency matrices	93.56	0.083	-
Our EEGNet	Spatial-spectral image Slab and spike representation	98.5	0.041	50.92

**Table 9:** Comparison accuracy for SVM, CNN, 3D CNN, multiview CGRN, and ssEEGNet.

Algorithms	DBN-3	GDBM-2	VGG-8	GoogleNet	ResNet-10	CDBN	ViT	Our EEGNet
Accuracy%	77.1	78.9	83.0	87.2	85.1	83.7	89.8	94.1

**Table 10:** Comparison accuracy for Ablation Model.

Ablation Model	Accuracy
Spatial-temporal EEGNet (dual-task learning and attention absent)	86.1%
Spatial-temporal EEGNet with attention (dual-task learning absent)	90.3%
Spatial-temporal EEGNet with dual-task learning (attention absent)	91.9%
Spatial-temporal EEGNet with dual-task learning and attention	94.1%

## Data Availability

All data included in this study are available upon request by contact with the corresponding author.
